# Coronaviruses as DNA Wannabes: A New Model for the Regulation of RNA Virus Replication Fidelity

**DOI:** 10.1371/journal.ppat.1003760

**Published:** 2013-12-05

**Authors:** Everett Clinton Smith, Mark R. Denison

**Affiliations:** 1 Department of Pediatrics, Vanderbilt University Medical Center, Nashville, Tennessee, United States of America; 2 The Elizabeth B. Lamb Center for Pediatric Research, Vanderbilt University Medical Center, Nashville, Tennessee, United States of America; 3 Department of Pathology, Microbiology and Immunology, Vanderbilt University Medical Center, Nashville, Tennessee, United States of America; Columbia University, United States of Amrica

## Coronaviruses Contain the Largest Known RNA Genomes

Coronaviruses (CoVs) are positive-sense single-stranded RNA viruses and contain the largest known RNA genomes, ranging from 27 to 32 kilobases in length. CoVs are capable of trans-species movement as evidenced by the Severe Acute Respiratory Syndrome coronavirus (SARS-CoV) epidemic in 2002–2003 [1], [2]. Additionally, the emergence of Middle East Respiratory Syndrome coronavirus (MERS-CoV) in 2012 demonstrates that CoVs continue to cause severe and lethal human disease [3]. Exactly how CoVs maintain the integrity of their large genomes while generating the population diversity required for emergence and adaptation has been a major question in RNA virology. The discovery of 3′-to-5′ exoribonuclease (ExoN) activity within CoV nonstructural protein 14 (nsp14-ExoN), which is critical for CoV high-fidelity replication, has challenged the long-held paradigm that RNA viruses cannot proofread and raises the possibility of an entirely new model for how RNA viruses regulate replication fidelity. We will summarize: 1) the data supporting proofreading during CoV replication; 2) the possibility of a multi-protein fidelity complex, using *E. coli* DNA polymerase III as a conceptual framework; and 3) the promise of genetic and therapeutic interference with fidelity regulation as an approach for attenuation and treatment of CoVs.

## CoVs Encode a Proofreading 3′-to-5′ Exoribonuclease Distinct from the Viral RNA-Dependent RNA Polymerase

RNA viruses rely primarily on low-fidelity replication by RNA-dependent RNA polymerases (RdRps) to facilitate viral adaptation to complex host environments [4]. Because of the lack of proofreading and repair functions, the average mutation rate of RNA viruses is estimated to be around one mutation per genome per round of replication [5], [6]. Much beyond this rate, RNA viruses risk crossing an “error threshold,” or the point at which there are too many deleterious mutations for the viral population to reproduce faithfully [4]. Thus, while allowing for enormous population diversity, the low-fidelity of RdRp-mediated replication imposes constraints on both viral genome size and maintenance of genomic integrity, theoretically limiting the size of RNA virus genomes to around ∼15 kb (reviewed in [4]). RNA viruses have evolved several mechanisms to partially circumvent these constraints, including large population sizes, rapid replication cycles, robustness to mutations, recombination, and compact genomes [7], [8].

**Figure 1:**
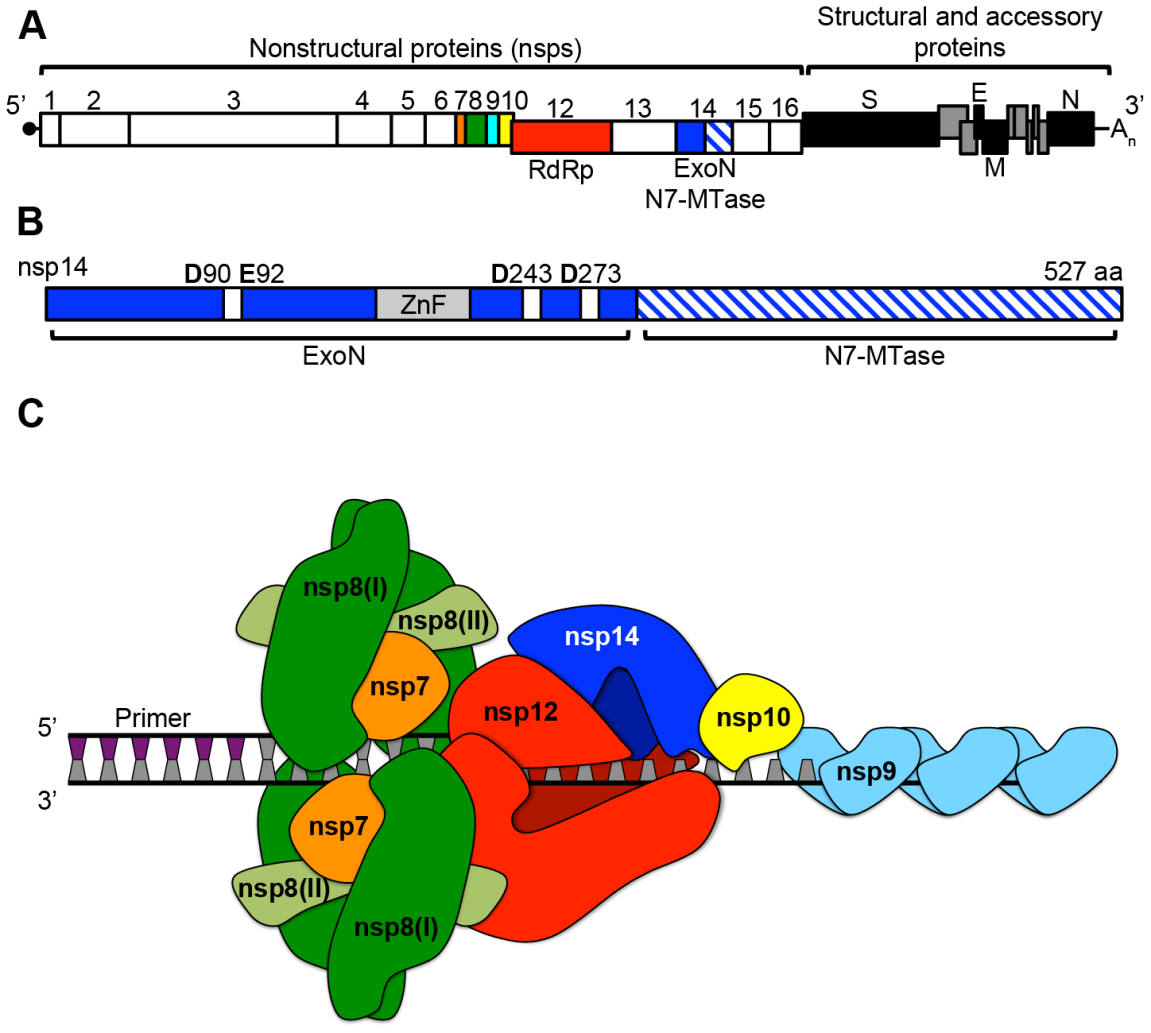
CoV genomic architecture and nonstructural proteins (nsps). **A.** Shown is a linear schematic of the SARS-CoV genome containing both the nonstructural protein (nsp) and the structural and accessory protein open reading frames. The −1 ribosomal frameshift between nsps 10 and 12 is shown by the offset boxes. Boxes denoting each individual nsp are scaled according to amino acid length, and products of polyprotein 1a (nsps1–10) and 1ab (nsps12–16) processing are shown. Colors are as follows: orange (nsp7), green (nsp8), cyan (nsp9), yellow (nsp10), red (nsp12: RNA-dependent RNA polymerase [RdRp]), and blue (nsp14: 3′-to-5′ exoribonuclease [ExoN] and N7-methyltranferase [N7-MTase]). **B.** A linear schematic of nsp14 is shown. The ExoN domain is colored solid blue, while the N7-MTase domain is hatched blue and white. The zinc-finger domain (ZnF) is shown in gray. DE-D-D residues characteristic of the DEDDh exonuclease superfamily [10] are shown as white boxes. **C.** A model of how nsps7–8 could assemble on viral dsRNA and interact with the putative multi-subunit CoV polymerase complex consisting of nsps10, 12, and 14. Colors are the same as in panel A, except the two forms of nsp8 (I and II) are shown in green and light green respectively. A short (∼6 n.t.) primer generated by the non-canonical RdRp activity of nsp8 is shown. Binding of ssRNA by nsp9 is also shown.

In addition to these mechanisms, CoVs encode several additional RNA-processing functions within their 16 nonstructural proteins (nsp1–16), including a 3′-to-5′ exoribonuclease (nsp14-ExoN; **Figure 1**). Many of these enzymatic functions likely have facilitated the expansion of the CoV genome beyond the ∼15 kb upper limit. CoV nsp14-ExoN contains four conserved DE-D-D acidic residues (**Figure 1B**; [9]), which are the hallmark of the DEDDh superfamily of DNA and RNA exonucleases. Many of these DEDDh exonucleases are involved in proofreading [10]. Alanine substitutions at the CoV DE-D-D residues significantly reduces or abolishes the nucleolytic activity of nsp14-ExoN *in vitro* [9] and results in CoVs with up to 20-fold reduced fidelity (increased mutation rate) *in vitro* [11], [12] and *in vivo* [13]. A recent biochemical study demonstrated that nsp14-ExoN is capable of removing single 3′ mismatched nucleotides and that this activity is stimulated by the non-enzymatic protein nsp10 [14]. Thus, all biochemical and genetic studies indicate that CoV nsp14-ExoN performs a proofreading function during CoV replication.

## Is nsp14-ExoN the Proofreading Component of a Multi-Subunit Polymerase Complex Containing nsp10 and nsp12-RdRp?

The identification of a proofreading exoribonuclease distinct from the CoV RNA-dependent RNA polymerase (nsp12-RdRp) suggests that CoVs might use a multi-protein complex for RNA synthesis and regulation of fidelity. A possible analogy would be the *E. coli* DNA polymerase III (pol III) core (αεθ), which contains a polymerase (α subunit), a DEDDh exonuclease (ε subunit), and an exonuclease stimulatory protein (θ subunit) [15]. Is there evidence to support or to even suggest the possibility that CoVs contain a DNA pol III–like multi-subunit polymerase complex? Several lines of evidence support the hypothesis that regulation of CoV fidelity involves, at minimum, three nsps: nsp12-RdRp, nsp14-ExoN, and nsp10 (**Figure 1C**). The presence of nsp12-RdRp would be critical as nsp14-ExoN has not been demonstrated to possess polymerase activity, and thus error recognition, removal, and repair would require interactions between nsp14-ExoN and nsp12-RdRp on elongating RNA. Several studies with poliovirus, chikungunya virus, and Coxsackievirus have demonstrated that point mutations within the viral RdRp can decrease or increase fidelity [16]–[19]. While the fidelity of nucleoside triphosphate (NTP) incorporation for nsp12-RdRp has not been determined, CoV nsp12-RdRp would provide essential polymerase activity and likely function to regulate fidelity primarily through NTP discrimination.

The CoV nsp10 is a ∼130 amino acid protein with no identified enzymatic function that has been demonstrated to bind and enhance nsp14-ExoN activity *in vitro* by up to 35-fold [14]. While the precise mechanism and function of this stimulation during virus replication remain unknown, nsp10 also plays a critical yet undefined role in viral polyprotein processing and RNA synthesis [20], [21]. Unlike prototypical members of the DEDDh exonuclease superfamily, nsp14-ExoN contains a zinc-finger (ZnF) domain that is likely critical for associating with viral RNA, though the affinity of nsp14 for RNA has not been determined (**Figure 1B**). Nsp10 also has two ZnF domains [22]. Thus, nsp10 might increase the affinity of nsp14 for RNA, or directly affect ExoN activity through allosteric mechanisms. Extending our analogy of CoV nsp10 to the theta (θ) subunit of the *E. coli* pol III core, theta is a ∼76 amino acid protein with no known enzymatic function that has been shown to both bind and stabilize the pol III ε subunit and stimulate ε-mediated removal of terminal mismatches [23], [24]. Thus, nsp10 could act to increase nsp14-ExoN stability, or possibly alter the capacity or preference of nsp14 to remove certain types of mismatches. Why CoVs encode an enhancer of ExoN activity remains unknown. However, the presence of such a complex might allow for greater control, and potentially active regulation, of replication fidelity.

## CoVs Encode Primase, Single-Strand RNA Binding, and Helicase Activities within nsps 8, 9 and 13

Might other CoV nonstructural proteins be associated with this putative multi-subunit polymerase? And how would such a complex be assembled? Again using *E. coli* pol III as a model, in addition to the αεθ core, the pol III holoenzyme requires additional subunits including a processivity factor (β sliding clamp), single-strand DNA-binding proteins (SSB), and a helicase (DnaB; reviewed in [15]). Remarkably, ssRNA-binding, helicase, and NTPase (*e.g.*, ATPase) functions have been identified within the CoV replicase. SARS-CoV nsp9 dimerizes and binds both ssDNA and ssRNA in a sequence-independent manner [25]. The capacity of nucleic acid to be bound by multiple nsp9 dimers strongly suggests that nsp9 could function as an SSB-like molecule, thus protecting the CoV genome from degradation during replication (**Figure 1C**). NTPase and helicase activity have been identified within nsp13 of human coronavirus 229E [26], [27]. Because dsRNA is likely an abundant replicative intermediate, nsp13 could function ahead of the polymerase complex and unwind the dsRNA, which would then be subsequently bound by nsp9. More recent work has shown that SARS-CoV nsp8 has non-canonical RdRp activity and likely functions as a primase [28]. Additionally, two distinct folds of nsp8, named I and II, participate in a hexadecameric nsp7-nsp8 supercomplex capable of primer extension and binding RNA, likely due to the presence of a ∼30 Å central channel lined with positively charged amino acids (**Figure 1C**) [29], [30]. Combined, these studies suggest that the nsp7-nsp8 supercomplex could be analogous to the β sliding clamp within the pol III holoenzyme, which increases both the processivity and speed of the α subunit [15].

## Is Fidelity Regulation a Target for CoV Inhibitors?

**Figure 2:**
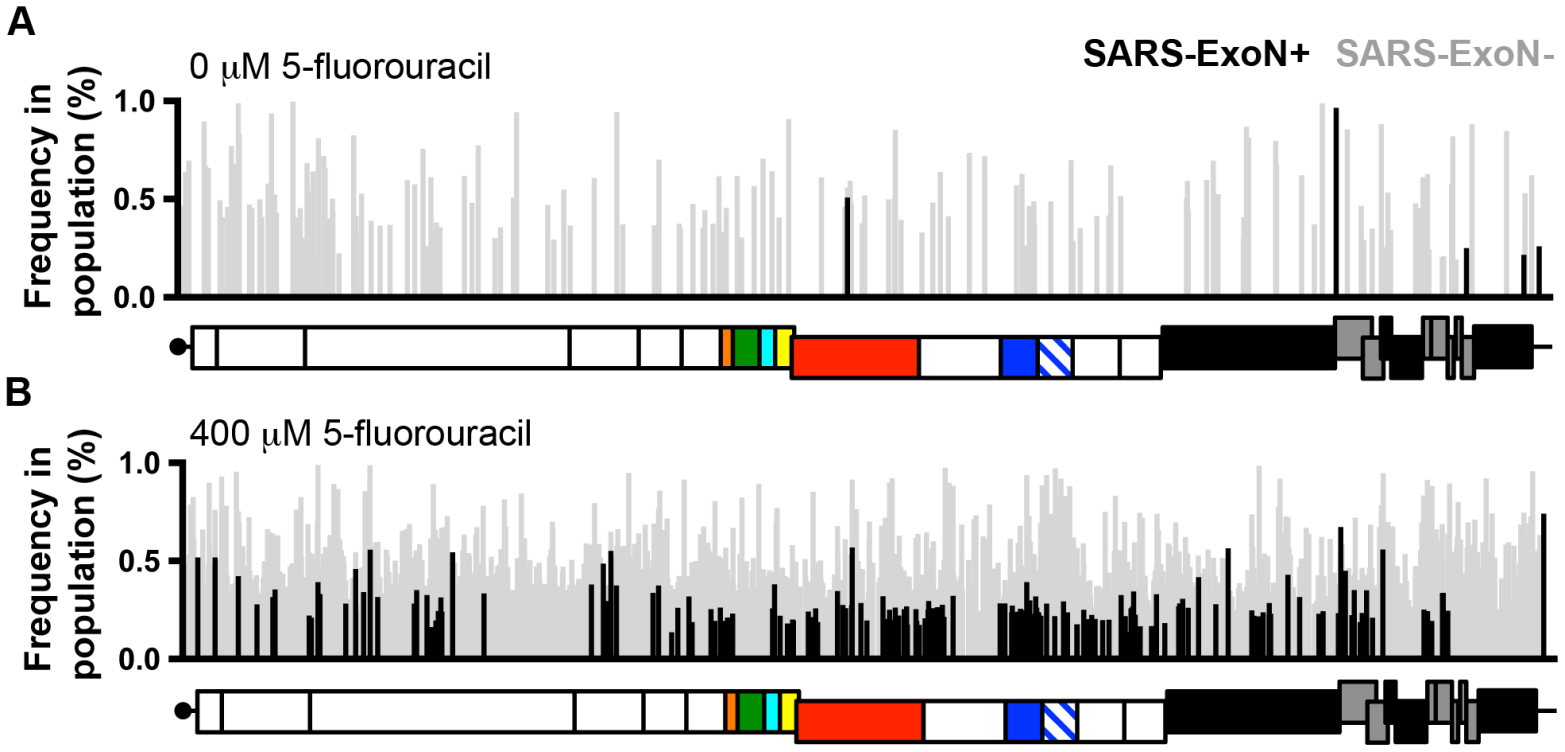
Loss of ExoN activity dramatically increases the sensitivity of CoVs to RNA mutagens. The distribution of characteristic 5-fluorouracil (5-FU)-mediated mutations (A-to-G and U-to-C) across the genomes of ExoN+ (wild-type; black) and ExoN− (gray) SARS-CoV following treatment with 0 µM (**A**) or 400 µM (**B**) 5-FU during single-cycle replication as determined by Illumnia deep sequencing. Each mutation is denoted as a vertical line, with line height representing the frequency of each mutation within the viral population. The genomic schematic below each panel show the approximate position of each mutation. Data are originally from [34].

Ribavirin (RBV) and 5-fluorouracil (5-FU) have been shown to be mutagenic for many RNA viruses (reviewed in [31]). Nucleoside analogs, including both mutagens and chain terminators, have been tested or are used to treat many RNA and DNA viruses, including herpesviruses, HIV, hepatitis C virus, and hepatitis B virus. However, *in vitro* and *in vivo* studies demonstrate the limited antiviral activity and variable effectiveness of RBV against SARS-CoV infection [32], [33]. Our recent study shows that nsp14-ExoN is responsible for CoV resistance to RNA mutagens. SARS-CoV and murine hepatitis virus (MHV) lacking ExoN activity (ExoN−) demonstrate 160- to 300-fold increased sensitivity to 5-FU [34]. While 5-FU treatment significantly increases the number of mutations present within both wild-type (ExoN+) and ExoN− viruses, almost 4,000 mutations were present in the ExoN− population following a single round of replication (**Figure 2**). This suggests that small-molecule inhibitors of ExoN could reproduce this genetic defect, and render CoVs vulnerable to treatment with RNA mutagens and obligate or non-obligate chain-terminating nucleoside analogs. The complete conservation of the ExoN− genotype and phenotype in CoVs studied to date [11]–[13], and the lack of redundancy or complementation of ExoN activity, further suggests that ExoN inhibition is a broadly applicable approach to therapeutically target CoVs. Furthermore, ExoN− viruses are highly attenuated *in vivo*, suggesting that small-molecule inhibition of ExoN activity would also significantly impact virus infection and virulence [13]. Finally, the probability that fidelity is regulated by a multi-protein complex involving nsp12-RdRp, nsp14-ExoN, and nsp10 suggests that interference with fidelity could be achieved through the disruption of multiple enzymatic activities and/or protein-protein interactions. The opportunity to disrupt the process of CoV fidelity regulation through the simultaneous targeting of multiple proteins, instead of a single protein or enzymatic function, has the potential for broad applicability and for potentially thwarting the emergence of resistance, particularly in combination with mutagens or other nucleoside analogs.

## Summary

Coronaviruses encode a proofreading exoribonuclease that is responsible for genome expansion, increased robustness to mutations, and resistance to mis-incorporations during RNA synthesis, as well as being required for virulence. The stability of the ExoN− genotype and phenotype provides a powerful model for the study of additional CoV fidelity determinants and of the effects of altered fidelity on virus replication, fitness, host-species range, and response to environmental changes. Experiments testing the proposed multi-subunit fidelity complex will yield exciting new insights into how CoVs ignore the RNA virus playbook and instead seem to play by their own rules while dancing on the edge of genetic disaster.
